# Biogeographic Patterns and Ecological Roles of Microorganisms in Sediments Along an Estuarine Salinity Gradient

**DOI:** 10.1111/1758-2229.70139

**Published:** 2025-07-02

**Authors:** Zongxiao Zhang, Guo Yuan, Xakila Turgun, Zulpinur Turgun, Lijun Hou, Mao Ye, Yonghui Wang, Xingbin Xu

**Affiliations:** ^1^ College of Geographic Science and Tourism, Xinjiang Normal University Urumqi China; ^2^ State Environmental Protection Key Laboratory of Integrated Surface Water–Groundwater Pollution Control School of Environmental Science and Engineering, Southern University of Science and Technology Shenzhen Guangdong China; ^3^ Technical Research Center for Environmental Geotechnical Engineering Restoration and Resource Utilization, Xinjiang Normal University Urumqi China; ^4^ State Key Laboratory of Estuarine and Coastal Research, East China Normal University Shanghai China

**Keywords:** assembly processes, bacteria composition, co‐occurrence patterns, estuarine sediment, nitrogen cycling

## Abstract

The distribution patterns and driving mechanisms of microbial biogeographic patterns are fundamental questions in microbiology. This study analysed and compared the bacterial biogeographic patterns in the coastal environment, focusing on the Yangtze Estuary and its adjacent coastal zone. The purpose is to explore the driving mechanisms under spatial distribution, the community assembly processes and potential functions. Our results revealed that the sediment bacterial community structure exhibited a distinct geographical pattern and was significantly influenced by environmental factors. The microbial community displayed a non‐random co‐occurrence pattern, and the biogeographic patterns were shaped not only by environmental constraints (deterministic processes) but also by stochastic processes resulting from dispersal limitation. The metagenome sequencing analysis revealed a pronounced salinity gradient in the nitrogen‐cycling function of the bacterial community. This functional difference appears to be driven by microbial diversity changes from the estuarine region to the ocean, highlighting the key role of microbial ecological characteristics. The findings of this study contribute to a deeper understanding of microbial ecology in estuarine environments, emphasizing the complex interplay between environmental factors and microbial community dynamics in shaping the function of estuarine sediment bacterial communities.

## Graphical abstract

From the river coastal to the adjacent sea waters, the diversity characteristics, community assembly processes, functional potential and community compositions of sedimentary microbial communities exhibit significant changes with increasing salinity levels. Microbial functional characteristics also show distinct variations along the salinity gradient and are influenced by environmental factors.

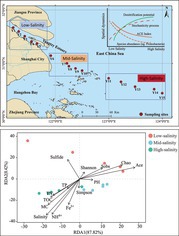

## 1 Introduction

Bacteria remarkably affect the redox reaction and the organic remineralization, thereby promoting the biogeochemical cycle (Sørensen 1982; Ducklow 2000). Particularly, in dynamic estuaries, as rivers bring in allochthonous substrates and particle resuspension increases, bacterial activity is enhanced by such intense chemical and physical processes (Santos et al. 2014). Organic matter as well as nutrients brought about by freshwater discharge, shallow sediments and the terrestrial environment enhance primary as well as secondary production in the nearshore environment. These organisms and resources are exported offshore, which increases the geographic effect imposed by highly productive zones out of the coastline (Fortunato et al. 2012; Satinsky et al. 2017). Estuary spanning brackish or freshwater and the marine environment makes the microbial community pattern complicated to interpret; hence, the salinity gradient usually dominates the formation of the microbial community pattern (Satinsky et al. 2017), demonstrating salinity as an essential component for influencing the microbial distribution structure (Lozupone and Knight 2007). Nevertheless, recent research suggests that microorganisms non‐randomly distributed in the coastal environment are also due to the geographic distance (Xiong et al. 2014; K. Wang et al. 2015). Spatial distance is capable of limiting microorganism spread as well as accelerating speciation (Diniz‐Filho and Telles 2000). In coastal oceans, there is some evidence showing a strong gradient in the nearshore to offshore microbial community composition, which might be shaped by distance, suggesting the unignorable influence of stochastic processes on the variation of microbial distribution (Fortunato et al. 2012; Z. Wang et al. 2019).

The Yangtze Estuary, together with the adjoining East China Sea (ECS) are dynamic areas that dramatically promote the global elemental exchange (Liu, Wu, et al. 2018). The regions harbour a complicated hydrological environment formed by the Taiwan warm current (south) together with the Yellow Sea coastal water (north) (Chen et al. 1999). Besides, the intertidal ecological system here is subjected to sewage discharge and pollutants from urban river runoff (Liu et al. 2001; Kuotung et al. 2009; Shi et al. 2014). More and more studies focus on the microbial ecology in these areas (Zhang and Jiao 2007; Feng et al. 2009; Sun et al. 2015; Liu et al. 2015; Ye et al. 2016; Guo et al. 2017). Yet, those studies paid more attention to the environmental factors affecting the marine microbial community and were less concerned with the deeper complicated promoting factors (such as assembly processes) for the microbial community pattern from nearshore to open ocean. Deterministic and stochastic processes were two different processes in the assembly of microbial communities, representing the niche and neutral perspectives in community ecology, separately (Hubbell 2001; Leibold and Mcpeek 2006). Microbial community assembly processes have been investigated in numerous habitats, and the contributions of these two processes for microbial assembly vary in different ecosystems (Jiao, Yang, et al. 2019; Liu et al. 2020). So far, we remain unclear about the balance of different assembly processes for the bacterial community in these regions. Microorganisms are capable of forming a complicated interaction network in a certain ecological niche (Faust and Raes 2012). Co‐occurrence network analysis can help us to better understand the structure possessed by a complicated microbial community as well as various functional interactions among microorganisms (Barberán et al. 2012). However, the bacterial co‐occurrence pattern exhibited by species in the sediments from the Yangtze River Estuary to the ECS remains unclear.

Additionally, we know less about the functional potential of bacteria in these places. In this century, global nitrogen (N) overload has been identified as a major environmental issue, due mainly to the widespread application of artificial N fertilizers and excessive combustion of fossil fuels (Gruber and Galloway 2008; Kim et al. 2014; Hou et al. 2015). Much of the anthropogenic N is delivered to the estuarine and coastal areas through river flow, groundwater discharge and atmospheric deposition (Seitzinger 2008; Diaz and Rosenberg 2008; Kim et al. 2014), consequently exerting a serious threat to these aquatic ecosystems. Microbially mediated N recycling processes, such as nitrification and dissimilatory nitrate reduction to ammonium (DNRA), occur simultaneously with the removal processes of denitrification and anaerobic ammonium oxidation (anammox) in estuarine sediments (Lisa et al. 2015). Therefore, an understanding of N removal processes and related microbial mechanisms is important for developing N management strategies to protect estuary ecosystems.

In this study, the 16S rRNA gene amplicon was adopted for analysing the community structure of bacteria, taking into account the environmental factors together with spatial factors. Then we evaluate the relative importance of species sorting and dispersal limitation in shaping sediment microbial communities across different salinity regions in estuary ecosystems. The null model was used to discern the relative importance of niche and neutral processes that structure community composition. Additionally, the co‐occurrence patterns exhibited by bacterial taxa in estuarine and coastal ecological systems were also explored. Meanwhile, to improve our understanding of the microbial N transformations in the estuarine and coastal ecosystems, metagenomic deep sequencing analysis was used to reveal N metabolic pathways along the estuarine salinity gradient.

## 2 Materials and Methods

### 2.1 Field Sampling

**FIGURE 1:**
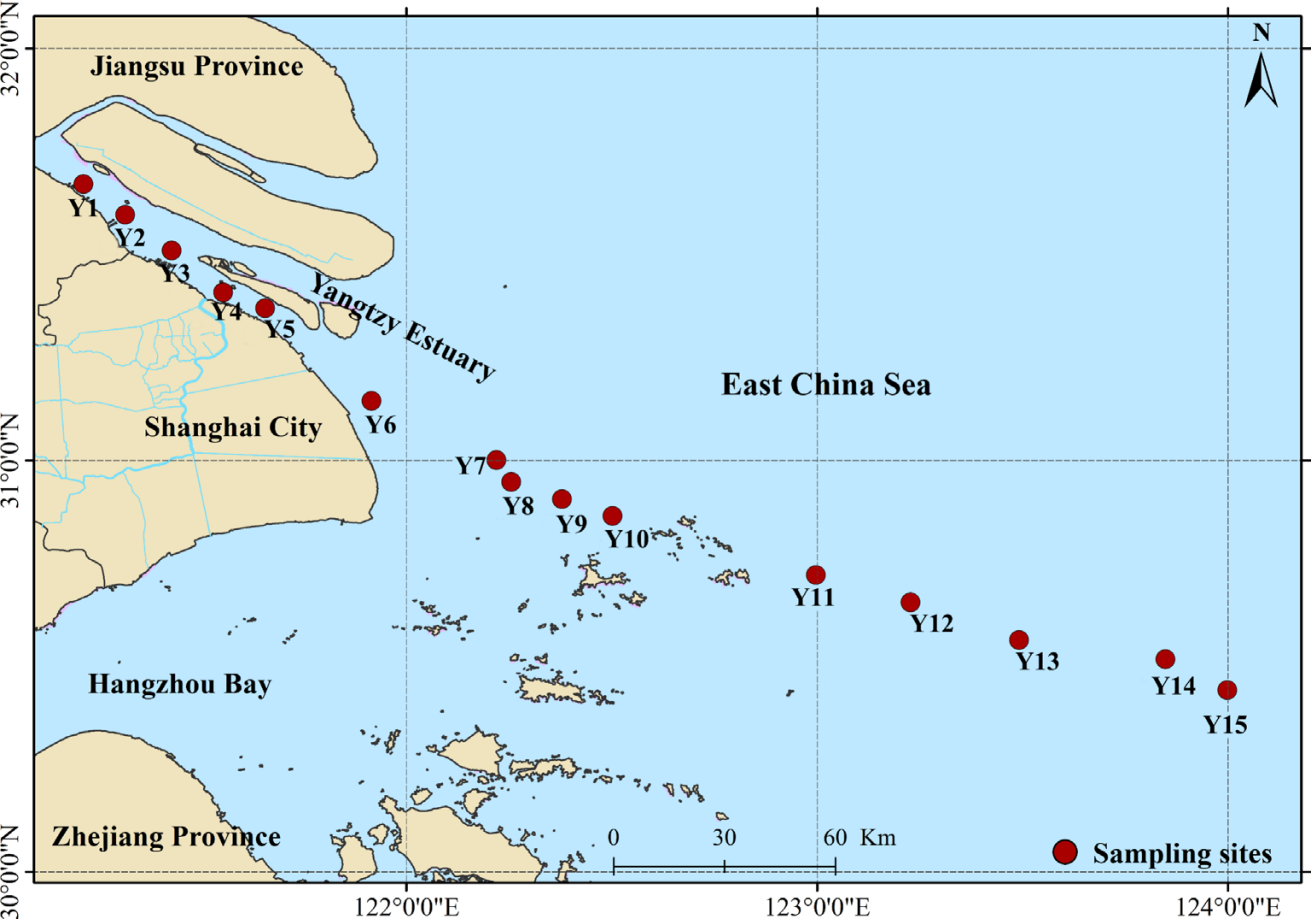
Study area. The figure shows the location of the Yangtze Estuary and the sampling sites during field investigations.

Fifteen sites (Y1–Y15) of the Yangtze River together with its adjoining coastal zones were selected as the study sites (Figure 1 and Table S1). The water depth in the study site was in the range of 5–60 m. There was an obvious salinity gradient (0.12–35.8 ppt) along the transect of samples. The salinity distribution was taken into account to divide these study sites into high‐salinity sites (32.9–35.8, Y11–Y15), mid‐salinity sites (8.72–26.8, Y6–Y10) and low‐salinity sites (0.12–0.98, Y1–Y5). We imported the global positioning system (GPS) coordinates that were recorded at every sampling point (from 121°12.825′ E 31°40.257′ N to 123°59.884′ E 30°26.484′ N) into the NOAA website (http://www.nhc.noaa.gov/gccalc.shtml) for calculating the pairwise geographic distance between samples. The surface sediment (0–5 cm) of each site was sampled in triplicate through subcoring a 50 cm × 50 cm × 50 cm box corer using PVC tubes (diameter: 7.2 cm; length: 5 cm), packaged samples in sealed sterile plastic bags added with dry ice in the cruise process, which were then transported to the lab and stored at −80°C.

### 2.2 Analyses of Environmental Characteristics

Table 1 lists the environmental parameters, which involved salinity, pH, exchangeable nitrite (${\mathrm{NO}}_{2}^{-}$‐N), nitrate (${\mathrm{NO}}_{3}^{-}$‐N), ammonium (${\mathrm{NH}}_{4}^{+}$‐N), Fe^2+^ and Fe^3+^ concentration, sulphide concentration, total organic carbon (TOC), moisture content (MC), total phosphorus (TP) and total nitrogen (TN). A portable water quality analyser (HQ 40d, HACH, USA) assisted in measuring the pH and salinity of a mixture of fresh sediments and deionised water free of CO_2_ at 1:2.5 (Zheng et al. 2014). Next, 2 M KCl was processed for the extraction of sediment inorganic nitrogen. Then, continuous‐flow injection analysis was used to measure the ${\mathrm{NH}}_{4}^{+}$, ${\mathrm{NO}}_{3}^{-}$ and ${\mathrm{NO}}_{2}^{-}$ concentration (Skalar Analytical SAN++, the Netherlands) (Hu et al. 2017). Measurement of Fe^2+^ and Fe^3+^ concentration followed the previous literature (Zheng et al. 2019), together with their references (Roden and Lovley 1993). The methylene blue spectrophotometric method assisted in analysing the sediment sulphide (Cline 1969). Vario EL CN Elemental Analyzer (Elementar, Germany) assisted in measuring the TOC acidified by 1 M HCl (Zhang et al. 2015). Water content of the sediment was determined by the weight loss of a certain amount of fresh sediment, which was dried at 80°C to a constant value (Zheng et al. 2016). After digesting sediments with the mixture of H_2_SO_4_–HClO_4_, we adopted the ascorbic acid‐molybdate blue method for assaying TP and applied a spectrophotometer to the measurement (Hou et al. 2013). Using 1 M HCl solution to leach the sediments, we then determined TN with a CN thermal combustion furnace analyser (Elementar Analyzer Vario Max CN, Germany) (H. Li et al. 2014). All of these physicochemical parameters were measured in triplicate and expressed as the TOC, TP and TN concentrations in dry weight.

**TABLE 1:** Physiochemical properties along the Yangtze Estuary to ECS zone.

Sampling sites	Salinity (ppt)	pH	^—^ ${\mathrm{NO}}_{2}^{-}$‐N (μg/g)	${\mathrm{NO}}_{3}^{-}$‐N (μg/g)	${\mathrm{NH}}_{4}^{+}$‐N (μg/g)	Fe^2+^ (mg/g)	Fe^3+^ (mg/g)	Sulphide (μg/g)	TOC (mg/kg)	MC (%)	TN (mg/kg)	TP (mg/kg)
Y1	0.12	8.35	0.612	8.81	11.27	1.05	0.70	0.01	16.26	40.59	1196.20	683.10
Y2	0.15	8.31	0.457	7.14	16.32	0.63	0.24	0.29	8.70	27.03	1523.10	781.90
Y3	0.56	8.16	0.413	8.52	17.22	0.35	0.06	0.13	7.14	27.59	1172.40	799.40
Y4	0.78	8.21	0.344	7.69	22.09	0.67	0.01	1.50	12.24	30.10	969.30	423.10
Y5	0.98	8.43	0.441	8.87	24.3	0.33	0.48	0.06	6.33	31.81	779.65	692.30
Y6	8.72	8.16	0.243	9.04	40.28	0.06	0.58	0.01	3.49	21.82	1082.00	1163.90
Y7	11.4	8.40	0.471	9.71	31.81	0.36	1.66	0.04	5.97	25.24	846.20	491.60
Y8	26.8	8.00	0.368	9.78	33.95	1.34	0.30	0.01	10.81	39.42	798.20	588.00
Y9	26.5	8.23	0.512	11.47	39.62	1.52	0.05	0.01	11.81	40.95	2698.60	733.60
Y10	26.7	8.11	0.242	3.44	40.26	1.13	0.57	0.01	12.00	39.13	1615.32	761.10
Y11	34.7	8.73	0.144	4.28	42.62	0.64	1.18	0.01	16.10	43.53	1682.00	450.90
Y12	33.8	8.18	0.225	7.54	39.40	0.90	1.02	0.01	15.22	47.44	841.20	966.00
Y13	32.9	8.40	0.137	1.72	53.77	1.32	0.70	0.02	14.25	49.22	2088.88	982.30
Y14	35.8	8.21	0.203	7.82	41.05	0.47	1.64	0.01	16.21	42.05	1084.10	747.10
Y15	35.4	8.16	0.261	7.99	40.33	1.14	1.08	0.50	19.20	49.93	4509.10	740.40

### 2.3 DNA Extraction and Purification

FastDNASPIN Kit (MP Biochemicals, Solon, OH, USA) assisted in extracting community DNA from sediment samples (0.5 g) in line with the protocol of the manufacturer. Primers 338F (5′‐ACT CCT ACG GGA GGC AGC AG‐3′) together with 806R (5′‐GGA CTA CHV GGG TWT CTA AT‐3′) were used to amplify the 16S rRNA gene V3‐V4 hypervariable region, modifying the forward primer to contain a unique 8 nt barcode at the 5′ end (Walters et al. 2016). PCR was performed in a 25 μL volume containing 12.5 μL 2 × Taq MasterMix, 1 μL of each primer (10 μM), 1 μL of template DNA and 10.5 μL of sterile water. The PCR program included an initial denaturation at 95°C for 3 min, followed by 30 cycles of denaturation at 95°C for 30 s, annealing at 55°C for 30 s and extension at 72°C for 30 s, with a final extension at 72°C for 5 min. We adopted the electrophoresis to detect PCR products in the bright band range of 400–450 bp in a 2% (w/v) agarose gel and mixed these PCR products in the same density ratio as well as purified them via GeneJET Gel Extraction Kit (Thermo Scientific) (Jiao et al. 2016). Libraries were constructed using the NEBNext Ultra DNA Library Prep Kit for Illumina (NEB, USA) according to the manufacturer's instructions. Library quality was assessed using the NanoPhotometer (IMPLEN, CA, USA) and Qubit dsDNA Assay Kit (Life Technologies, CA, USA). Library quantification was performed using the KAPA Library Quantification Kit (KAPA Biosystems, USA). Subsequently, we mixed three parallel PCR products in each subsample to sequence on an Illumina platform. Sequencing was performed on the Illumina MiSeq platform in PE300 (paired‐end 300 bp) mode.

### 2.4 16S rRNA Amplicon Sequence Analysis

FLASH (V1.2.7, http://ccb.jhu.edu/software/FLASH/) assisted in merging the paired‐end reads, and the quality of reads was filtered based on the literature (Magoc and Salzberg 2011). The USEARCH software was applied to the detection and removal of acquired sequences following the UCHIME algorithm (Edgar et al. 2011). These sequences had their barcodes during the assignment to each sample. The UPARSE software package was applied to the sequence analysis by the UPARSE‐OTU algorithm and UPARSE‐OTUref algorithm. We assigned sequences with a similarity of 97% to the same OTU. The RDP classifier assisted in assigning the sequences representing each OTU to the taxonomic groups (Caporaso et al. 2011).

### 2.5 Metagenome Sequencing and Statistical Analysis

For each sample, we placed 120 ng of genomic DNA in 60 μL of buffer (10 mM Tris–HCl, pH 8.0) into 1.5 mL RNase‐/DNasefree, microcentrifuge tubes with low binding. A NanoPhotometer spectrophotometer (IMPLEN, CA, USA), together with a Qubit dsDNA Assay Kit in a Qubit 2.0 Flurometer (Life Technologies, CA, USA), was applied to analyse quality. NEBNext UltraTM DNA Library Prep Kit for Illumina (NEB, USA) assisted in preparing the DNA library by using 1 mg of DNA in line with the recommendation of the manufacturer. For each sample, the attribute sequence was added with an index code. AMPure XP system helped purify these samples, and an Agilent 2100 Bioanalyzer (Agilent Technologies, CA, USA) helped to measure the libraries. A cBot Cluster Generation System was adopted to generate a cluster, and then paired‐end reads (PE150) were carried out on an Illumina HiSeq 2500 platform to generate a 10‐Gb metagenomic data set.

Then we filtered the paired‐end reads with low quality (quality score ≤ 38; base *N* > 10 bp, and overlap length between adapter and reads > 15 bp). SOAP de novo v.2.21 helped to assemble the sequences (Luo et al. 2012), and K‐mer sizes of 49, 55 and 59 were selected as the scaffolds containing the largest N50. Scaffolds with lengths over 500 bp were reserved as well as produced via scaffold fragmentation for the following analysis. About assembled metagenomes, MetaGeneMark v.2.10 helped to predict the open reading frames (Gemayel et al. 2022), CD‐HIT v.4.5.8 (Li and Godzik 2006) helped to build a non‐redundant gene catalogue (unigenes) and SoapAligner v.2.21 helped to control the quality (eliminating the genes with < 3 reads per sample) (Gu et al. 2013). DIAMOND (Buchfink et al. 2015) with the help of the KEGG database and NR database (BLASTp, *e*‐value ≤ 1e − 5) achieved the functional annotation and taxonomy. KEGG Orthologs (KOs) were identified by aligning the gene sequences to the KEGG database. These KO assignments allow for the classification of gene functions into specific pathways and broader functional categories. The KOs were assigned to higher‐level KEGG categories and specific KEGG pathways.

### 2.6 Null Model and Network Analysis

Null model analysis was carried out using the framework described by Stegen et al. (2013) to classify community pairs into underlying drivers of deterministic process and stochastic process based on the β‐nearest taxon index (βNTI). And the stochastic processes, including homogenizing dispersal (mass effect), dispersal limitation and drift, were distinguished by Raup‐Crick metric (RCbray) as described by Liu et al. (2020). The null model expectation was generated using 999 randomizations. To explore the co‐occurrence patterns of bacterial taxa, we selected the top 100 OTUs based on their relative abundance across all samples. Specifically, we calculated the sum of relative abundances for each OTU across all samples and selected the 100 OTUs with the highest summed relative abundances. A Spearman's correlation between two OTUs presented statistical robustness with a coefficient over 0.6 and the *p*‐value less than 0.01 (Barberán et al. 2012; Jiao et al. 2016). The interactive platform Gephi assisted in visualizing the network (Bastian et al. 2009).

### 2.7 Data Analyses

QIIME (http://qiime.org/index.html) was employed for calculating the Alpha and Beta diversity, adopting 3581 reads/sample (i.e., the minimum sequence number needed for normalizing the sequencing depth difference), with multiple indices and the Bray‐Curtis distance between samples. The differences in physicochemical properties and community diversity between groups were calculated based on two‐way analysis of variance (ANOVA), and the differences in taxonomic community (based on 16S sequences) and functional potential (based on metagenome sequences) abundances among salinity groups were compared via Kruskal–Wallis *H* test, respectively.

We confirmed the relation of geographical coordinates or the environmental factor (the Euclidean distance based on environmental factors) with the sample ordination on the taxonomic (Bray–Curtis). The Mantel test assisted in checking the effect of environmental and spatial dissimilarity on taxonomy. Nonmetric multidimensional scaling (NMDS) and principal coordinates analysis (PCoA) were used for evaluating the structure and function difference of microbial community (Caporaso et al. 2010; Legendre and Legendre 1998).

Two‐way analysis was used in comparing the spatial variations of environmental variables. Based on the values of lengths of gradient, we applied the canonical correspondence analysis (CCA) and redundancy analysis (RDA) for examining the relationships between microbial taxonomic and functional structure and environmental indices in Canoco (version 4.5) software, respectively (Danovaro and Gambi 2002). The linear trend together with the principal coordinates of neighbour matrices (PCNM) procedure assisted in deriving the spatial variable from the geographic coordinates (Griffith and Peresneto 2006), thereby minimizing the association of spatial distance with environmental factor, and capturing all spatial scales detected in the dataset. The adjusted *R* ^2^ was used in the analysis of variation partitioning for determining the different effects of spatial factor and environmental factor on the bacterial community variation, as well as the combined effect of the two factors.

N‐cycling gene family refers to the parent category of N‐cycle function that was defined in the KEGG “Nitrogen metabolism” module (denitrification, nitrification, N‐fixation, Comammox, Assimilation and Dissimilation nitrate reduction), as well as the gene family defined by the KOs. Additionally, to accurately assess the relative abundance of functional genes, we employed the RPKM (Reads Per Kilobase of transcript per Million mapped reads) method, which could more accurately compare the relative abundance of nitrogen‐cycling genes across different samples, ensuring that the results are not biased by gene length and sequencing depth.

Except as described above, the rest of the data analysis processing is done in the R environment (http://www.r‐project.org). The raw reads of metagenomic and high‐throughput sequences have been deposited in the NCBI SRA database under the accession number PRJNA642351.

## 3 Results

### 3.1 Geochemical Characteristics of Sediments

Table 1 lists the major physicochemical characteristics and geographic characteristics of the sampling site. All sediments were alkaline, and pH ranged from 8.00 to 8.73. Sediment nutrient factors, ${\mathrm{NO}}_{2}^{-}$ and ${\mathrm{NH}}_{4}^{+}$ showed significant variations among three salinity levels (low, mid and high) as defined in the methods section (two‐way ANOVA, *R* ^2^ = 0.607, *p* = 0.008 and *R* ^2^ = 0.848, *p* < 0.001). Likewise, significant variations were observed for the concentrations of sediment TOC (two‐way ANOVA, *R* ^2^ = 0.524, *p* = 0.003), with values of 3.49 mg/kg in mid‐salinity sites to 19.20 mg/kg in high‐salinity sites. The values of MC were in the range of 21.82%–49.93% with relatively higher contents at high‐salinity sites (*R* ^2^ = 0.574, *p* < 0.001). Significant variations were observed for Fe^3+^ concentrations (two‐way ANOVA, *R* ^2^ = 0.428, *p* = 0.022), whereas they had no variations for Fe^2+^ concentrations (two‐way ANOVA, *F* = 0.648, *p* = 0.540).

### 3.2 Diversity and Richness of Bacterial Communities

The Miseq sequencing platform assisted in identifying the structure of the bacterial community in all sediments. We obtained a data set containing 53,715 quality sequences from the 15 samples. The total OTU number was 3189, defined by 97% sequence similarity. Table 2 lists the bacterial community abundance and diversity in sediments along the salinity gradient in the Yangtze Estuary to the ECS zone. Regarding the bacterial diversity exhibited by sediments, the Shannon indices were in the range of 3.10–5.97, and the highest values were seen at low‐salinity sites Y2. Chao 1 and Aces indices ranged from 626.33 to 1382.95 and 782.01 to 1455.78, respectively. Moreover, the highest values of these two richness indices also appear at low‐salinity sites Y2. According to the results of the alpha diversity index, bacterial communities at low‐salinity sites presented relatively higher diversity and richness, with Shannon, Chao 1 and Ace's index averaged at 5.43, 1171.86 and 1106.76, respectively. The results of the two‐way ANOVA indicate that only the Ace richness estimate shows a significant difference among different salinity groups (*R* ^2^ = 0.504, *p* = 0.015), suggesting that the Ace index has distinct variations across salinity levels. Other indices, such as Sobs, Shannon, Simpson, Chao 1 and Coverage, did not reach significant levels (*p* > 0.05), implying that the variations in these indices among different groups may be more influenced by other random factors.

**TABLE 2:** Diversity characteristics of bacterial communities along the Yangtze estuary to ECS zone.

Sampling sites	Sobs	Shannon	Simpson	Chao 1	Ace	Coverage
Y1	909	5.90	0.01	1272.86	1357.59	0.89
Y2	943	5.97	0.01	1382.95	1455.78	0.88
Y3	684	5.19	0.03	947.92	1005.67	0.92
Y4	622	4.79	0.03	949.80	1021.41	0.92
Y5	676	5.30	0.02	980.29	1018.85	0.92
Y6	471	4.25	0.06	854.21	1250.61	0.93
Y7	273	3.10	0.13	626.33	849.03	0.96
Y8	829	5.80	0.01	1182.03	1268.27	0.90
Y9	720	5.33	0.02	1057.59	1150.67	0.91
Y10	776	5.42	0.02	1125.02	1212.00	0.90
Y11	547	4.45	0.08	764.38	808.46	0.94
Y12	667	5.28	0.02	898.66	925.32	0.93
Y13	647	5.60	0.01	818.20	860.01	0.94
Y14	485	3.72	0.11	698.19	782.01	0.94
Y15	642	5.60	0.01	827.31	864.02	0.94

### 3.3 Bacterial Geographic Patterns Along the Salinity Gradient

Most sequences (98.11%) were included in the phylum of bacteria. There were mainly 6 phyla of Proteobacteria, Chloroflexi, Bacteroidetes, Epsilonbacteraeota, Acidobacteria and Nitrospirae (relative abundance > 4%), occupying 73.11% of the total sequences. Also, Planctomycetes, Actinobacteria, Firmicutes, Fusobacteria and Verrucomicrobia could be found in the majority of samples, and their relative abundances were lower (Figure S1).

**FIGURE 2:**
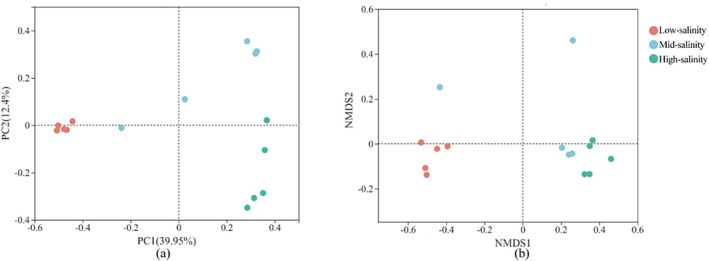
The (a) principal coordinates analysis (PCoA) and (b) nonmetric multidimensional scaling (NMDS) analysis of the bacterial community revealed by the 16S rRNA gene sequences.

As revealed by PCoA and NMDS analysis, the bacterial community distribution exhibited a distinct geographical specificity along the salinity gradient of the Yangtze Estuary to the ECS zone. According to the first two axes of PCoA, there were three groups of bacterial assemblages along the salinity gradient (Figure 2a). Bacterial communities at the sites Y1–Y5 were classified into the group with low salinity. Bacterial communities at sites Y11–Y15 were classified into the group with high salinity, where we saw the relatively higher abundance of Epsilonbacteraeota, Planctomycetes and Fusobacteria (Figure S2). The remaining bacterial communities (at the sites Y6–Y10), where the mixing of seawater and freshwater was observed, were in the group with mid‐salinity, in which Bacteroidetes and Firmicutes showed relatively higher abundance (Figure S2). In addition, considering the OTUs that were found within samples, NMDS ordination analysis also obviously demonstrated the bacterial community differentiation (Figure 2b). An analysis of similarity made a further confirmation of these patterns, ANOSIM result indicating the vital significance of sampling sites for determining the community composition (*r* = 0.7636, *p* = 0.001). Based on all these results, bacterial composition presented a dynamic spatial pattern along the salinity gradient.

**FIGURE 3:**
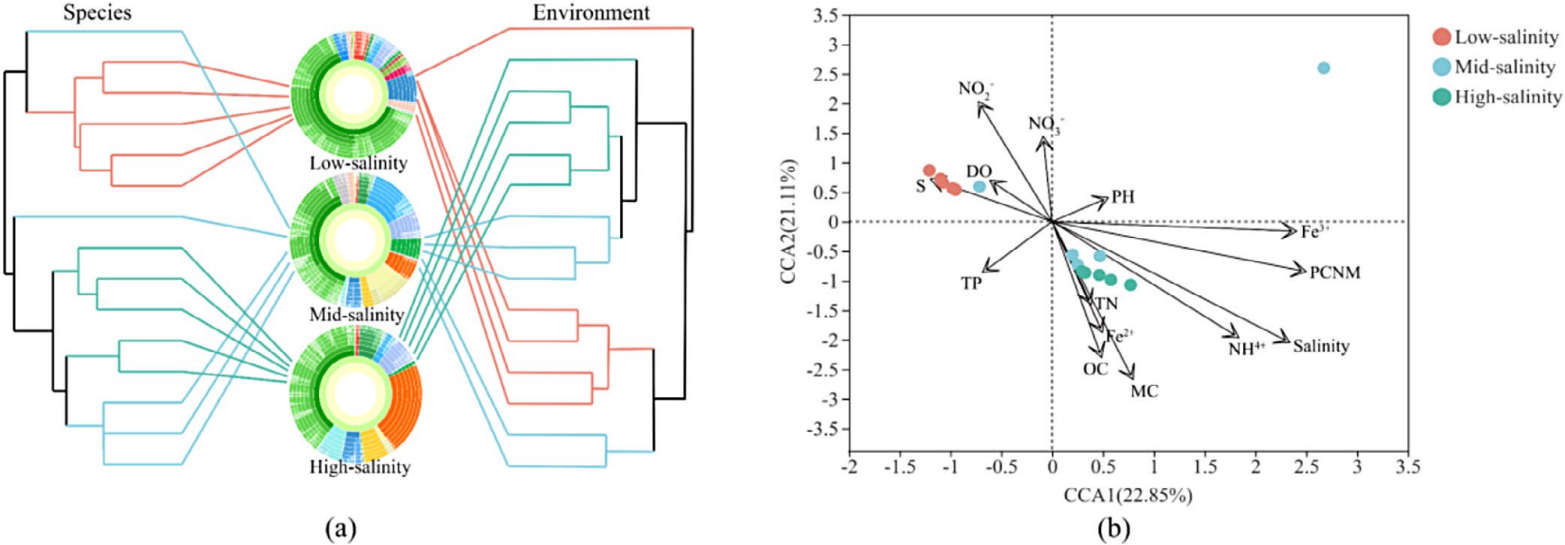
Dendrogram (a) of Bray–Curtis distance based on bacterial communities and Euclid distance based on environmental factors, branches are coloured by the sample sites that they represent; Sunburst charts showing the hierarchical taxonomic composition of a salinity group. The outer ring represents the OTU level, whereas higher taxonomic ranks are more closer to the centre. The colours are assigned automatically to distinguish taxa. (b) Canonical correspondence analysis (CCA) biplot of the distribution of bacterial communities with environmental factors.

Figure 3 displays the impact of environmental factors on the structure of the bacterial community along various salinity gradients. The clusters based on soil environment factor in the right diagram showed a similar cluster to the bacterial community structure in the left diagram, confirming the obvious association between them (the mantel test, *r* = 0.7392, *p* = 0.001) (Figure 3a). CAA analysis assisted in exploring the effect of environmental and spatial factor on bacterial community (Figure 3b). The first two CCA axes accounted for 43.96% of the cumulative variation regarding the relation between them. In which, bacterial community distribution and structure were affected by salinity (*p* = 0.001), ${\mathrm{NO}}_{2}^{-}$ (*p* = 0.029), ${\mathrm{NH}}_{4}^{+}$ (*p* = 0.003), Fe^3+^ concentration (*p* = 0.008), TOC (*p* = 0.015), MC (*p* = 0.001) as well as spatial factors (PCNM, *p* = 0.006). The variance partitioning analysis (VPA) was further used to quantify the effect of environmental and spatial factors on the variation of the bacterial community. The effect of the two factors accounted for 40.2% of the bacterial community variation. The pure effect of the environmental factor explained 8.6% of the variation. While the PCNM explained 1.6% of the variation. Also, the combined effect of PCNM and environmental variables accounted for 30.0% of the variation, demonstrating the mutual dependence of the two factors.

### 3.4 Bacterial Assembly Process and Bacterial Interactions

**FIGURE 4:**
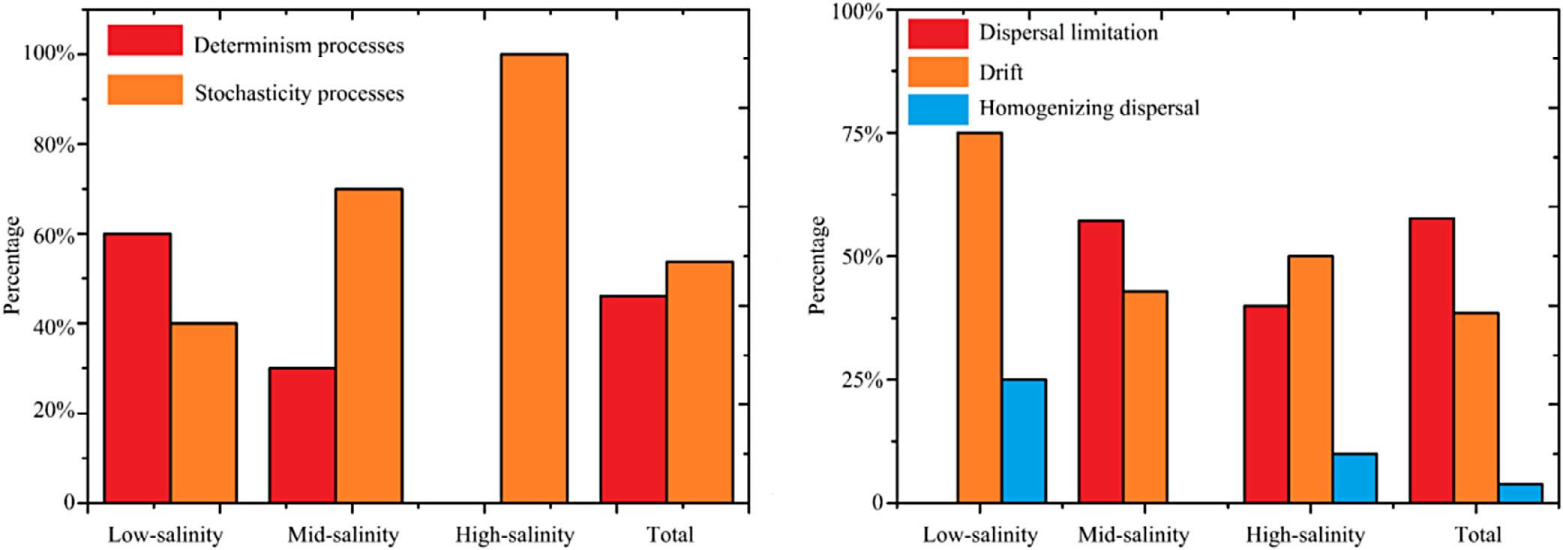
Null model analysis revealing the assembly mechanism of the bacterial community in salinity gradient and all samples (total). Left: the percent of determinism and stochasticity process. Right: the contribution of different stochastic processes that include homogenizing dispersal, dispersal limitation, and drift.

To explore community assembly mechanisms under the observed geographic pattern, the relative roles of niche and neutral processes in community assembly were analysed. Across all samples, stochastic processes explained a slightly higher proportion than deterministic processes of the bacterial community variation and the balance of these ecological processes varied greatly across the salinity gradient. The deterministic processes explained a higher proportion of the bacterial community variation than stochastic processes in the low‐salinity area, while stochastic processes were higher in the mid‐salinity site. Particularly, in the high‐salinity range, all the βNTI values between the sample points falling in the range of −2 to 2 (represent the stochastic process explained 100% of the bacterial community variation) (Figure 4). For the stochastic processes, the result showed that dispersal limitation was the most important process, accounting for 57.69% of the community variation across all samples, followed by drift, which explained 38.46% of the total variation, and the homogenizing dispersal only explained 3.85% of the total (Figure 4).

**FIGURE 5:**
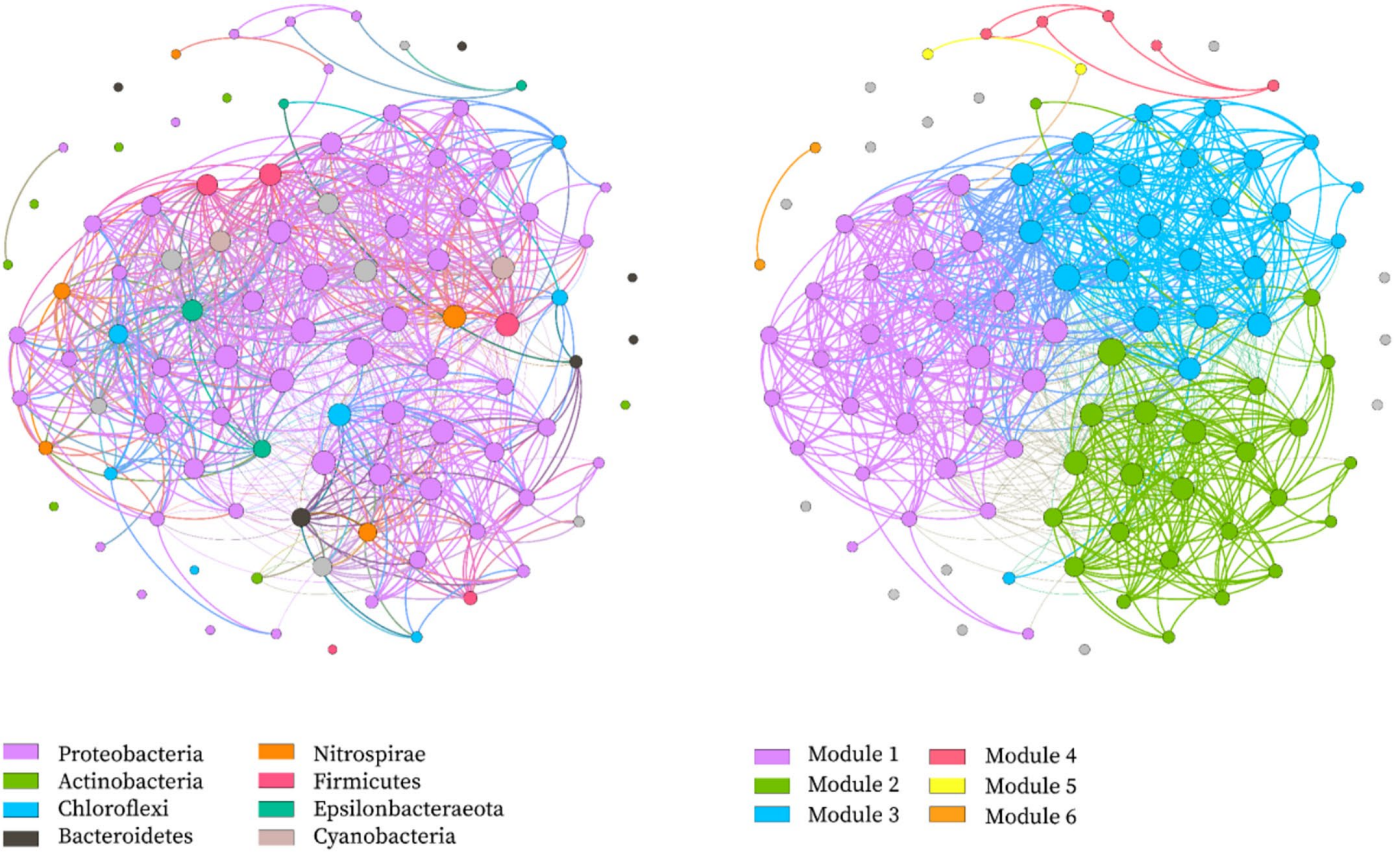
Network of co‐occurring bacterial OTUs based on correlation analysis. A connection stands for a strong (Spearman's *r* > 0.6) and significant (*p* < 0.01) correlation. The size of each node is proportional to the relative abundance; the thickness of each connection between two nodes (edge) is proportional to the value of Spearman's correlation coefficients. Left: co‐occurring network coloured by phylum. Right: co‐occurring network coloured by modularity class. The taxonomic and modularity characteristics of each node are detailed in Table S3.

The sediment microbial network contains 100 nodes (OTUs) together with 878 edges, and each node has 17.56 edges on average (average degree). It has an average path length (APL) of 2.003, and the diameter is 6. The clustering coefficient (CC) and modularity index (MD) were 0.654 and 0.779, respectively (values over 0.4 mean the existence of a modular structure in the network). The nodes in the network belonged to 11 bacterial phyla, with 8 widely distributed phyla (Proteobacteria, Bacteroidetes, Chloroflexi, Actinobacteria, Nitrospirae, Firmicutes, Epsilonbacteraeota and Cyanobacteria), which occupied 95% of the total. With node distribution being modularized, all nodes mainly fell into six modules (Figure 5). Those from Module 1 were largely the dominant OTUs in low‐salinity sites; nodes from Module 2 were mostly dominant OTUs in mid‐salinity sites; Module 3 mostly consisted of the nodes from OTUs in high‐salinity sites (Figure S3). Accordingly, the network structure exhibited an obvious salinity gradient pattern.

### 3.5 Potential Metabolic Pathways of Nitrogen Cycling

**FIGURE 6:**
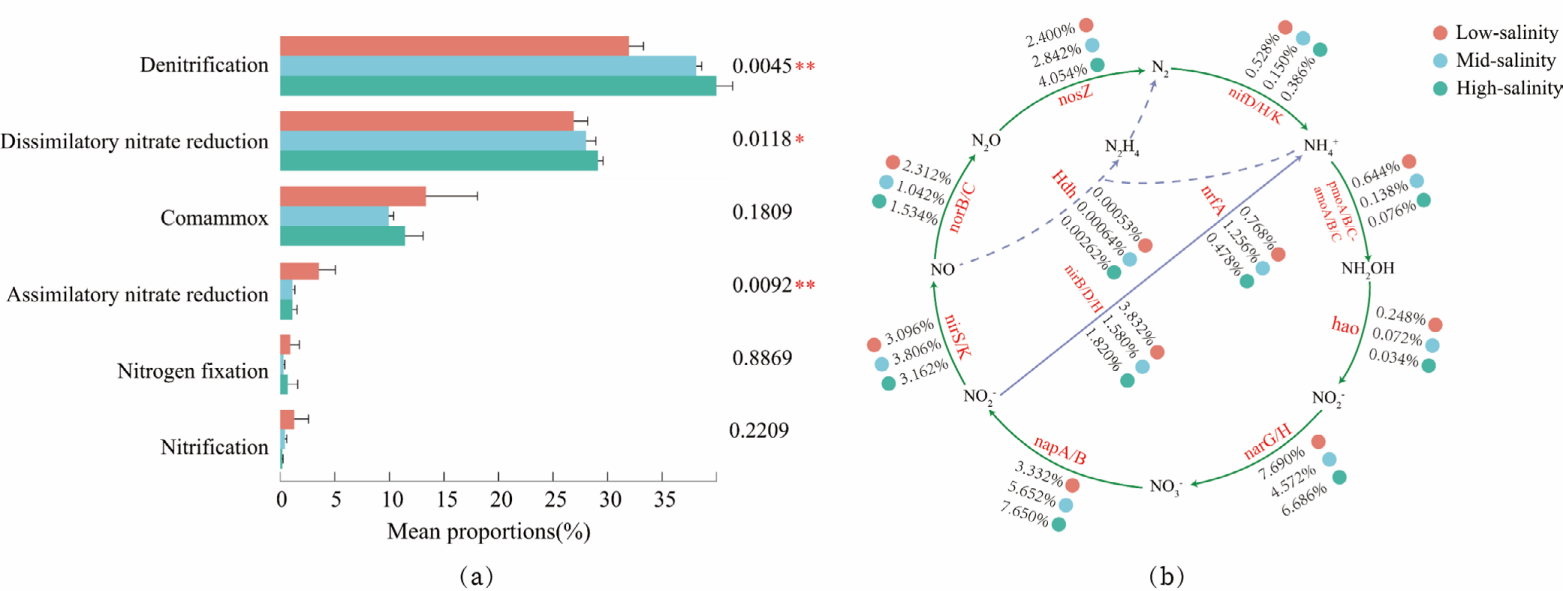
Genetic potential for N metabolism in different salinity sediment. (a) The statistical comparison of different KEGG pathway (Level 3, modules level) involved in nitrogen cycles. (b) Relative abundance of genes associated with nitrogen cycle. The mean proportion shown represents the relative abundance of nitrogen‐cycling genes calculated using the RPKM method.

A total of 6,502,591 nonredundant genes were detected in the metagenomes, and a total annotated 7576 KOs were annotated in the bacterial community. For nitrogen metabolism, 44 KOs were annotated to N metabolism, which covered the majority of the whole N metabolic pathway (Table S2). The predominance of dissimilatory nitrate reduction, denitrification and comammox was evident according to KEGG annotations. And the relative abundance of genes involved in dissimilatory nitrate reduction, denitrification, was more abundant in high‐salinity sediments compared to low‐ and middle‐salinity sites (Figure 6a). The high frequency of sequences affiliated with the genes *narG/H* and *napA/B* involved in nitrite/nitrate transformation was detected. Additionally, the genes involved in denitrification (*nirS/K*, *norB/C* and *norZ*) and dissimilatory nitrate reduction to ammonium (*nirB/D/H* and *nrfA*) also with high potential. In contrast, sequences related to N fixation (*nif D/H/K*) and nitrification (*hao* and *pmoA/B/C*‐*amoA/B/C*) showed low abundance. And the genes related to the anammox (*Hds*) were hardly detected (Figure 6b). The relative abundance of functional genes involved in nitrogen cycling was also varied among different salinities. The *napA/B*, *nirB/D/H* and *nosZ* genes were significantly more abundant in high‐salinity sites, and the *norB/C* and *Hdh* genes were more abundant in low‐salinity sites compared to high salinity regions (Figure 6b).

### 3.6 Effects of Bacterial Communities and Environmental Factors on Nitrogen Transformation

**FIGURE 7:**
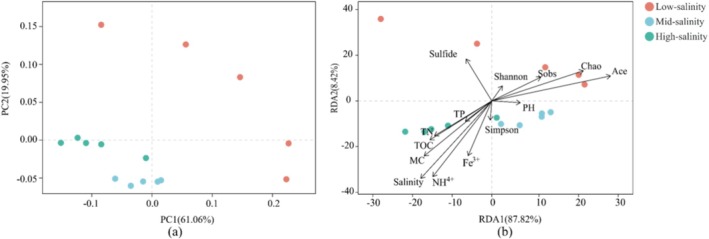
(a) Composition of functional genes (KOs level) involved in nitrogen cycle based on the principal coordinates analysis (PCoA) and (b) redundancy analysis (RDA) of the distribution of bacterial nitrogen cycle functional genes with environmental and microbiological factors.

The functional patterns of nitrogen metabolic genes (Figure 7a) formed the similar pattern as the bacterial community structure (Figure 2a). RDA was used to explore environmental and biological effect on the observed functional pattern (Figure 7b). The RDA result showed that three environmental factors (salinity, ${\mathrm{NH}}_{4}^{+}$ content and MC) and two microbiological factors (Chao 1 and ACE) were the most powerful predictors separating functional patterns along the salinity gradients (*p* < 0.05). And the VPA result appeared to show a higher effect of microbial factors than environmental factors, which accounted for 26.0% and 13.0% of the total variation, respectively. In addition, according to fitting analyses, significant correlations were confirmed between salinity and functional β‐diversity (*p* = 0.001, *R* ^2^ = 0.55), as well as bacterial richness (Chao 1 index) and functional β‐diversity (*p* = 0.015, *R* ^2^ = 0.38).

## 4 Discussion

The study focuses on discussing the characterization of the bacterial community and the functional profiles of nitrogen cycling from the Changjiang Estuary to the ECS along the salinity gradient. Additionally, this study pays attention to the microbial interaction and assembly process in the sediment ecological system with strong connectedness.

### 4.1 Important Factors Influencing Sediment Bacterial Community Structure

The bacterial communities were under the dominant by the phyla of Proteobacteria, Chloroflexi, Bacteroidetes, Epsilonbacteraeota, Nitrospirae and Acidobacteria. This pattern presents a similarity to the patterns of other marine sediments around the world (Fortunato et al. 2012; K. Wang et al. 2015; Z. Wang et al. 2019; Guo et al. 2018; Liu, Zhang, et al. 2018). As the sampling sites saw the enrichment of N, it was assumed that there were abundant bacterial taxa in the N‐cycle. To be specific, all the known nitrite‐oxidizing bacteria, which catalyse the second nitrification step and are the main process in the biogeochemical N‐cycle, are categorized into either the Proteobacteria or the genus Nitrospira of the phylum Nitrospirae (Daims et al. 2016), as well as Chloroflexi (Sorokin et al. 2012) were abundant in this research. Epsilonproteobacteria are often dominant in deep‐sea environments (Stokke et al. 2015), tending to prefer high‐salinity conditions. Acidobacteria have been found in a variety of habitats, including terrestrial and marine environments (McReynolds et al. 2025). Our study shows that Acidobacteria have higher abundance in low‐salinity areas, which may be due to greater terrestrial input in these areas, although our data are not sufficient to fully confirm this. Bacteroidetes are often attached to particles, degrading the polymeric substances (Fernández‐Gomez et al. 2013). Freshwater discharge converges with tidal current in the middle‐salinity site, forming an energetic area where accumulated sediments are resuspended to the water column (Shi et al. 2014), thus higher abundance of Bacteroidetes in the middle‐salinity site reveals their niches as well as life strategies.

From the Yangtze River Estuary to the ECS, the spatial differentiation exhibited by samples presented an obvious correlation with the salinity, and it seems that these factors pose a dramatic impact on bacterial community composition. As shown in several studies of the coastal zone, salinity mainly affects the bacterial diversity variation (Herlemann et al. 2011; Fortunato et al. 2012; K. Wang et al. 2015; Liu, Wu, et al. 2018). Salinity, as an environmental factor, mainly determines the community composition in soil, sediment, freshwater and marine environments, and exerts a more important effect compared with pH, temperature and so forth (Lozupone and Knight 2007). Besides, regarding the coastal environment, salinity is likely to cause a density gradient that separates the residential bacterial community in water masses (Fortunato et al. 2012). Here, we also found the importance of nutrient‐related variables (primarily TOC, ${\mathrm{NO}}_{2}^{-}$ and ${\mathrm{NH}}_{4}^{+}$), and MC also affected the bacterial community. Nutrients remarkably affect the composition and growth of bacteria in the environment, including nitrogen, carbon and iron in the bioavailable form, which may be fully utilized by microorganisms and closely related to the composition of the bacterial community (Hou et al. 2015; Soares et al. 2016). Studies also investigated the value of MC for the structuring of bacterial community composition, as it had a clear effect on the bacteria shown by Niederberger et al. (2008, 2015). In conclusion, a deterministic process (environment filtering) assisted in shaping the bacterial community from the Yangtze Estuary to the ECS.

### 4.2 Co‐Occurrence Patterns of Microorganisms

A microbial community sees the occurrence of complicated interactions, which could be reflected in the co‐occurrence networks to some extent (Faust and Raes 2012; Liu et al. 2019). The bacterial communities exhibited a connectedness as well as a non‐random co‐occurrence, which further demonstrated the impact of the deterministic process on the community structure. In addition, the modular structure was significantly subjected to salinity, possibly because of the co‐occurrence of dominant taxa with other taxa that had the same requirement for habitat (Delgado‐Baquerizo et al. 2018). Functions possessed by nodes in different modules may vary (Newman 2006; Jiao et al. 2016), and taxa that are strongly linked ecologically tend to cluster into the same modules. Thus, the community compositions and ecological functions interconnected along the Yangtze Estuary to the ECS zone might have potential relationships. Also, it is necessary to pay attention to the fact that the estimated co‐occurrence pattern variation obtained by using a system method based on topology was incapable of reflecting the actual correlation among taxa (Ma et al. 2016).

### 4.3 The Ecological Processes Governing the Microbial Communities' Assembly

In river estuary and its adjacent areas, water flow together with tidal activity mainly dominates the bacteria spread as well as the bacteria transportation to the benthic zone (Augspurger et al. 2010). Therefore, because the dispersal rate (mass effect) is high, bacterial composition may be randomly distributed within this highly connected ecological system (Mouquet and Loreau 2003; Van der Gucht et al. 2007; Liu, Zhang, et al. 2018). On that account, geographic distance may slightly affect the taxon diversification in the zone. Nonetheless, based on the null model, a stronger role of stochastic processes rather than deterministic processes in governing the bacterial community assembly was detected. For the high‐salinity samples, the null model revealed that stochastic processes were overwhelming. In contrast, in a low‐salinity site, the influence of stochastic processes was weak. A potential explanation is that high‐salinity sediment has a lower regional connectivity than low‐salinity areas (Zinger et al. 2014). In the present study, the deep‐sea sediments with relatively uniform environments (Jacob et al. 2013) allow stochasticity processes to dominate the community assembly. In systems with less environmental variation, the stochastic processes may overwhelm deterministic processes (J. Wang et al. 2013; Jiao, Xu, et al. 2019).

Here, we found that the stochasticity of the benthic bacterial community was mainly controlled by dispersal limitation and drift. The result is consistent with Wu and Huang (2019) reported that dispersal limitation accounted for 33.3% of the protist community turnover in sediments of the South China Sea, whereas drift provided an explanatory power of 13.3%, and homogeneous dispersal explained 0%. In addition, these indicators have obvious dynamic changes along the salinity gradient. The influences of dispersal limitation were almost non‐existent in low‐salinity sites, which might be due to the strong runoff effect of the Yangtze River, allowing sediment bacterial communities to disperse randomly. Therefore, environmental gradients (such as salinity gradients) and environmental conditions (such as hydrological conditions) might determine the balance of determinism and stochasticity, and thus construct the geographical pattern of bacterial community structure from the Yangtze Estuary to the ECS.

### 4.4 N Biogeochemical Processes

We further reconstructed microbial N biogeochemical processes in these salinity gradient regions based on the metagenomes. The genes were ranked according to their relative abundances. Nitrogen plays a key role in biogeochemical processes (Ollivier et al. 2011) and involves various microbe‐derived enzymes (Gruber and Galloway 2008). Therefore, the microorganisms involved in the process of nitrogen cycling (nitrogen fixation, nitrification, denitrification, DNRA and nitrogen assimilation) in estuarine sediment might respond to environmental factors.

Compared to the DNRA, genetic potential for denitrification was enriched in Yangtze Estuary sediment bacteria. And both of them have the same pattern of distribution (low‐salinity > mid‐salinity > high‐salinity). The DNRA process can retain nitrogen in the system in a bioavailable form (${\mathrm{NH}}_{4}^{+}$) for further biological processes, which would promote a positive feedback of eutrophication (Jäntti and Hietanen 2012). While denitrification is the process of reductive respiration and uses nitrate or nitrite to nitric oxide or nitrous oxide and then to nitrogen, which could remove nitrate “permanently” from aquatic ecosystems (Crowe et al. 2012). The higher abundance genes of DNRA may lead to more inorganic nitrogen from external sources being retained in this eutrophic estuarine ecosystem. Thus, most external inorganic nitrogen might remain retained in the Yangtze Estuary, which might be a possible factor causing severe eutrophication and frequent occurrence of harmful algal blooms (H. Li et al. 2014). It has been reported that potential rates of denitrification and DNRA in the sediments of the Yangtze Estuary contributed 38%–96% and 3%–45% total nitrate reduction, respectively (Deng et al. 2015). Which is consistent with the result based on the metagenome in this study.

Additionally, in this study, spatial gradients were found in bacterial community nitrogen cycling functional genes. This suggests the environmental gradient in this study area affects the functional pattern of nitrogen cycling. Furthermore, considering that nitrogen cycling pathways in sediments are mediated by a series of microorganisms that occupy different taxonomic levels (Marcel et al. 2018), the taxonomic characteristics change might affect the potential of nitrogen cycling functions. Here, our results showed that the bacterial diversity characteristics were the key factor affecting the spatial pattern of nitrogen cycle function, compared with the environmental factors. This suggests the undeniable influences of microorganisms in the nitrogen transformation in the estuary ecosystem. Furthermore, although further research is needed, we suggest that the balance of niche and neutral processes within microbial communities might influence their functional characteristics.

To sum up, based on the study results, the spatial distribution exhibited by the bacterial community and function is subject to the direct control of environmental factors and the indirect control of stochastic genetic drift and dispersal limitation. A future study is suggested to conduct sampling in time series and at least on the seasonal scale, for predicting dynamic bacterial assembly patterns on a time scale. It is necessary to integrate more detailed physicochemical information as well as hydrologic conditions with the functional information and taxonomy of microbes, for better characterizing the biogeographic community distribution as well as the functional structure. As metagenomic results do not reflect true abundance and are not always expressed in proportion, the annotated gene abundance seems to demonstrate the potential biogeochemical process instead of the microbiome‐mediated actual biogeochemical process. Multi‐omic profiling is needed to gain holistic insights into the genetic potential and main elements (e.g., nitrogen, sulphur and carbon) metabolic coupling of marine sediment microbial communities in future research. Anyway, our study provides an overall and new insight into microbial diversity and functionality in this coastal ecosystem. This might provide essential basic data for the protection and restoration of estuarine ecosystems.

## Author Contributions

**Zongxiao Zhang:** conceptualization, methodology, software, investigation, formal analysis, supervision, funding acquisition, visualization. **Guo Yuan:** resources, writing – original draft, writing – review and editing, data curation. **Xakila Turgun:** methodology, formal analysis. **Zulpinur Turgun:** data curation, investigation, writing – review and editing. **Lijun Hou:** writing – review and editing, resources. **Mao Ye:** supervision, formal analysis, resources. **Yonghui Wang:** data curation, investigation, writing – review and editing. **Xingbin Xu:** writing – review and editing, funding acquisition.

## Conflicts of Interest

The authors declare no conflicts of interest.

## Supporting information


Data S1.

## Data Availability

Sequence data were deposited in the National Center for Biotechnology Information (NCBI) Sequence Read Archive with BioProject PRJNA642351. Other data are available from the corresponding author upon reasonable request.
