# Supplementary material for: Biogeographic Patterns and Ecological Roles of Microorganisms in Sediments Along an Estuarine Salinity Gradient
Source: Environ Microbiol Rep. 2025 Jul 2;17(4):e70139. doi: 10.1111/1758-2229.70139 (PMC12221337; doi:10.1111/1758-2229.70139)
Supplement: Supplementary file 1 — Data S1. [file EMI4-17-e70139-s001.docx]

**Supplementary information**

**Biogeographic Patterns and Ecological Roles of Microorganisms in Sediments along an Estuarine Salinity Gradient**

Zongxiao Zhang^1,2,3*†^, Guo Yuan^1†^, Xakila Turgun^1^, Zulpinur Turgun^1^, Lijun Hou^4^, Mao Ye^1^, Yonghui Wang^1^, Xingbin Xu^1*^

^1^ College of Geography and Tourism, Xinjiang Normal University, Urumqi, 830054, China

^2^ State Environmental Protection Key Laboratory of Integrated Surface Water-Groundwater Pollution Control, School of Environmental Science and Engineering, Southern University of Science and Technology, Shenzhen, Guangdong 518055, China

^3^ Technical Research Center for Environmental Geotechnical Engineering Restoration and Resource Utilization, Xinjiang Normal University, Urumqi 830054, China

^4^ State Key Laboratory of Estuarine and Coastal Research, East China Normal University, Shanghai 200241, China

**†** These authors have contributed equally to this work

***** Corresponding author: [107622024010031@xjnu.edu.cn](mailto:107622024010031@xjnu.edu.cncom), and xuxingbin0214@126.com


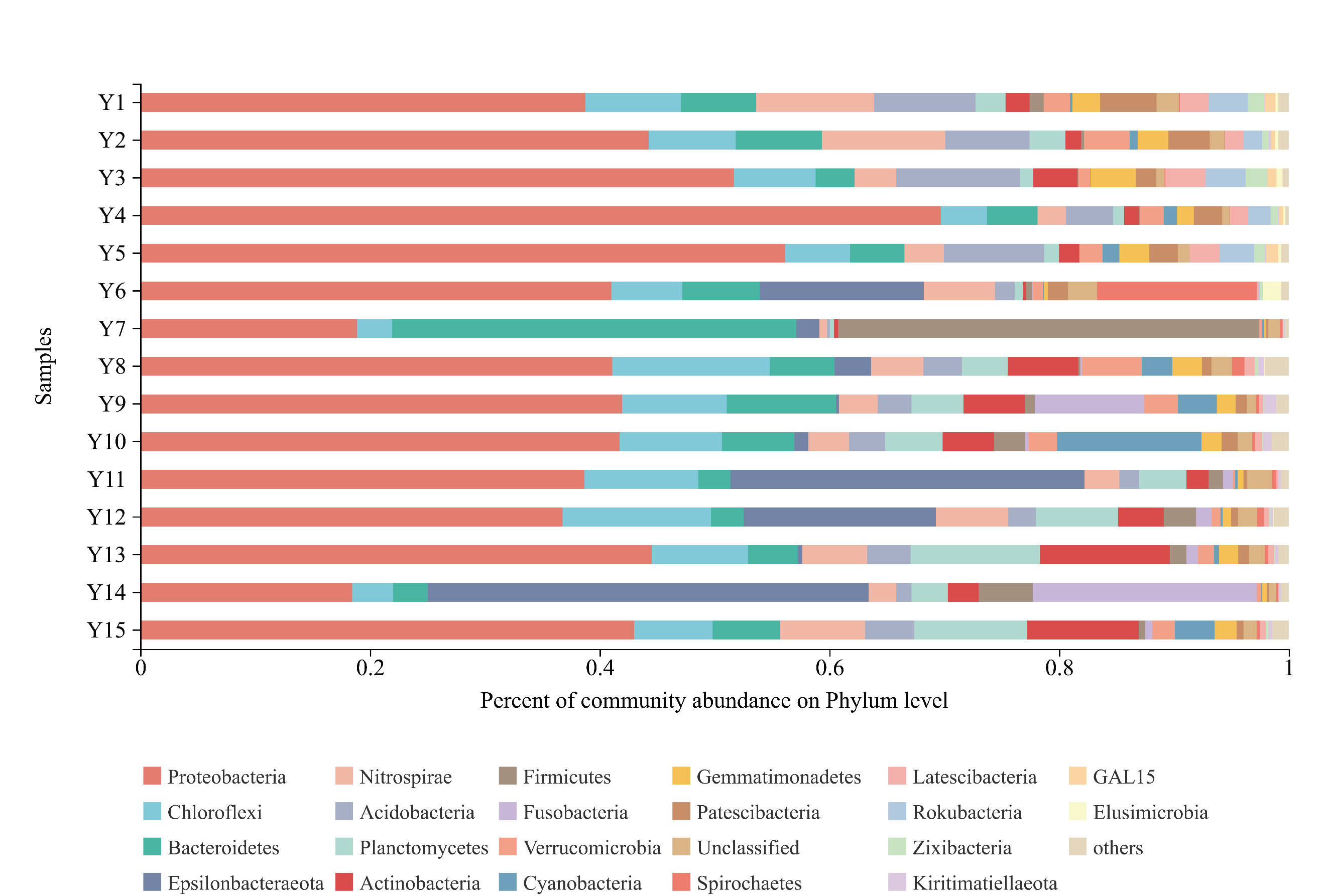


**Fig. S1.** Comparison of the relative abundance of bacterial sequences classified at the phylum.


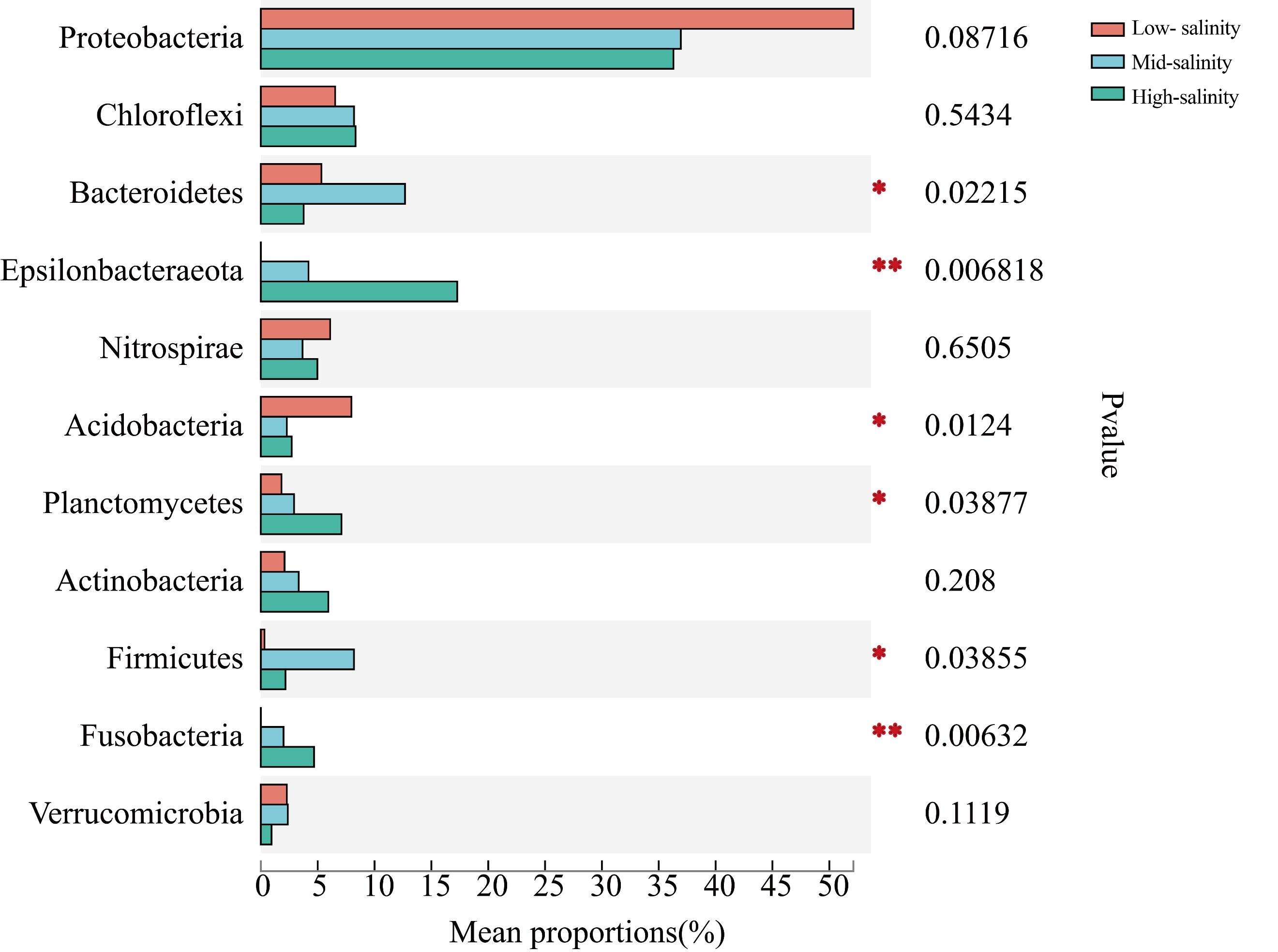


**Fig. S2.** Statistical comparison of the relative abundance of dominant phyla in the Low, Mid, and High-salinity groups. Significant differences are shown as follows: **P < 0.01 and * P < 0.05.


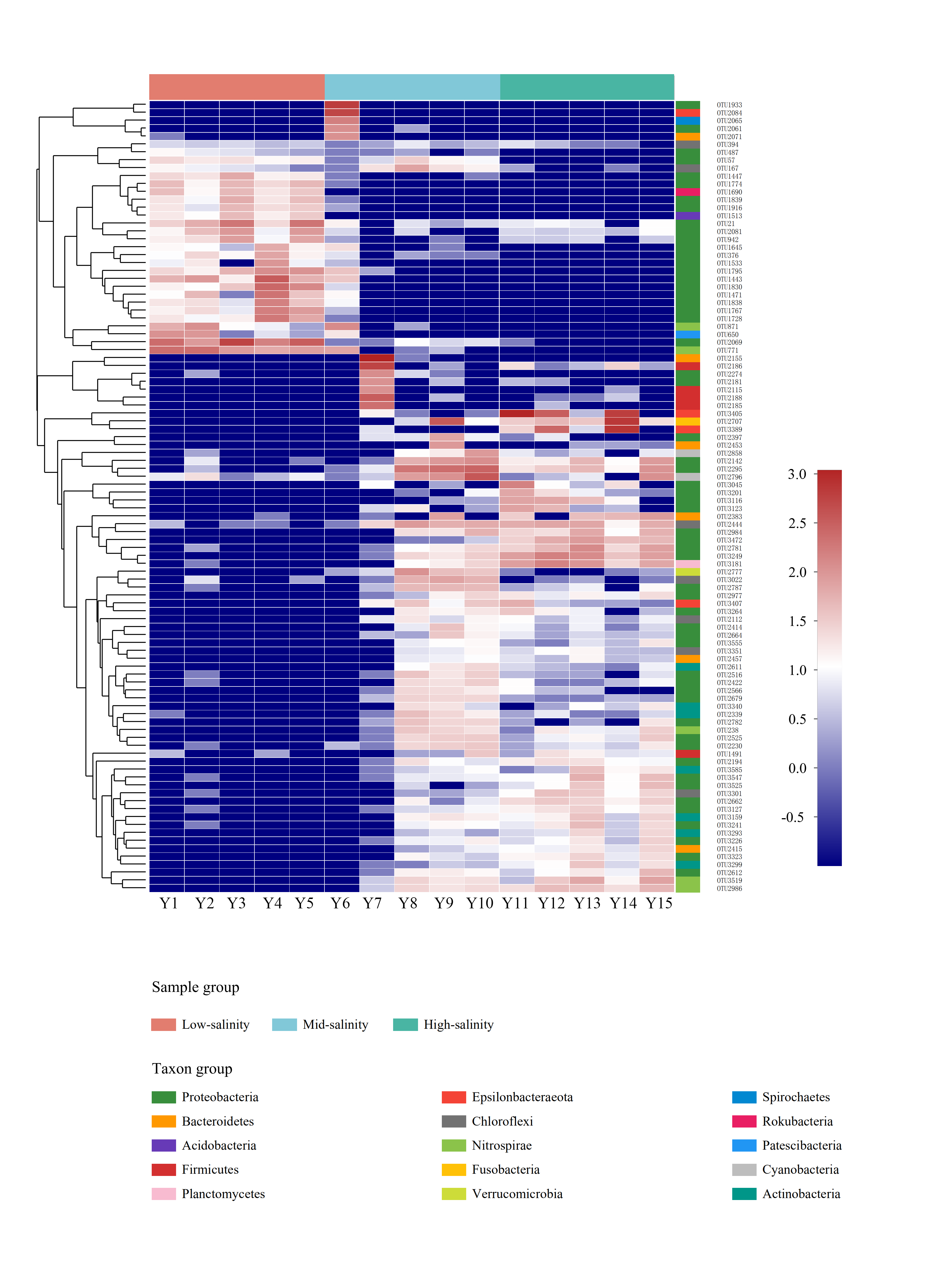


**Fig. S3.** Clustering of the top 100 most abundant OTUs in low, mid, and high-salinity sediment samples, OTUs in the same taxon (phylum) are marked with the same colors. Heatmap shows the abundance of the top 100 OTUs by a color gradient, after log10 transformation.

Table S1. Sample location, sampling time, and water depth in this study area

| Sampling | Sampling | Arrival | Depth | Longitude | Latitude |
| --- | --- | --- | --- | --- | --- |
| sites | Date | time | （m） | (°E) | (°N) |
| Y1 | 2019/7/14 | 16:34 | 12.8 | 121°12.825 | 31°40.257 |
| Y2 | 2019/7/14 | 15:31 | 19.4 | 121°18.948 | 31°35.790 |
| Y3 | 2019/7/14 | 14:25 | 13.9 | 121°25.724 | 31°30.574 |
| Y4 | 2019/7/14 | 13:14 | 11.4 | 121°33.238 | 31°24.481 |
| Y5 | 2019/7/14 | 12:30 | 11.6 | 121°39.380 | 31°22.174 |
| Y6 | 2019/7/14 | 10:30 | 10.1 | 121°54.916 | 31°08.659 |
| Y7 | 2019/7/14 | 8:32 | 10.2 | 122°13.152 | 31°00.036 |
| Y8 | 2019/7/14 | 7:59 | 10.6 | 122°15.309 | 30°56.839 |
| Y9 | 2019/7/14 | 6:34 | 12.5 | 122°22.748 | 30°54.309 |
| Y10 | 2019/7/16 | 5:30 | 16.3 | 122°30.113 | 30°51.871 |
| Y11 | 2019/7/16 | 9:28 | 48.0 | 122°59.836 | 30°43.270 |
| Y12 | 2019/7/16 | 11:13 | 58.6 | 123°13.641 | 30°39.279 |
| Y13 | 2019/7/16 | 13:05 | 52.8 | 123°29.498 | 30°33.772 |
| Y14 | 2019/7/16 | 15:17 | 48.1 | 123°50.868 | 30°30.991 |
| Y15 | 2019/7/16 | 16:33 | 47.2 | 123°59.884 | 30°26.484 |

Table S2. The relative abundance of key N metabolic functional genes according to KEGG annotations.

| KO | Annotated genes | Y1 | Y2 | Y3 | Y4 | Y5 | Y6 | Y7 | Y8 | Y9 | Y10 | Y11 | Y12 | Y14 | Y13 | Y15 |
| --- | --- | --- | --- | --- | --- | --- | --- | --- | --- | --- | --- | --- | --- | --- | --- | --- |
| K10946 | pmoC-amoC | 8.562E-05 | 0.0002686 | 0.00926 | 0.0076605 | 0.0022542 | 0.0010319 | 0.0005708 | 0.0005989 | 0.0004788 | 0.0007468 | 4.227E-05 | 0.000303 | 0.0002045 | 0.0002822 | 0.0004128 |
| K10945 | pmoB-amoB | 0 | 5.469E-05 | 0.003252 | 0.0034978 | 0.0018403 | 0.0003007 | 0.00054 | 0.0001986 | 0.0002752 | 0.0006334 | 0.0002069 | 9.388E-05 | 0.0002583 | 0.0004822 | 0.0004858 |
| K10944 | pmoA-amoA | 0.0002696 | 1.826E-05 | 0.0012299 | 0.0024514 | 6.375E-05 | 0.0004009 | 0.0002775 | 0.0002784 | 0.0001348 | 0.000399 | 7.624E-05 | 0.0001527 | 0.0001004 | 0.0002868 | 0.0004442 |
| K15578 | nrtC | 0.0059498 | 0.0098843 | 0.0067612 | 0.0094089 | 0.0145142 | 0.001368 | 0.0012382 | 0.0011709 | 0.0013071 | 0.001146 | 0.0022301 | 0.0016921 | 0.0019132 | 0.0010983 | 0.0001996 |
| K15577 | nrtB | 0.004676 | 0.0082199 | 0.0060339 | 0.0084455 | 0.0162829 | 0.0015432 | 0.0020073 | 0.0023935 | 0.0009148 | 0.0014154 | 0.002598 | 0.002324 | 0.0023866 | 0.0011342 | 0.0003316 |
| K15576 | nrtA | 0.0081209 | 0.0153176 | 0.0103932 | 0.0110358 | 0.0257927 | 0.001512 | 0.0025344 | 0.0028847 | 0.0015037 | 0.002051 | 0.0035847 | 0.0025396 | 0.0011654 | 0.0013981 | 0.0003751 |
| K15876 | nrfH | 0.0057657 | 0.0061923 | 0.0017068 | 0.0019439 | 0.0048405 | 0.0049288 | 0.0059317 | 0.0056226 | 0.0052225 | 0.0052086 | 0.0018688 | 0.0015396 | 0.0023098 | 0.0018585 | 0.0020172 |
| K02575 | NRT | 0.0251413 | 0.0353791 | 0.037194 | 0.0492331 | 0.0407189 | 0.0182137 | 0.0168232 | 0.0192362 | 0.0196975 | 0.022162 | 0.0358654 | 0.0297353 | 0.028372 | 0.0277876 | 0.0250226 |
| K03385 | nrfA | 0.0120592 | 0.0097421 | 0.0065927 | 0.0041501 | 0.0058635 | 0.0118181 | 0.0140638 | 0.0118508 | 0.0117445 | 0.013279 | 0.0043381 | 0.0044185 | 0.0063274 | 0.0037034 | 0.0050825 |
| K02305 | norC | 0.010117 | 0.0107166 | 0.0021285 | 0.0059601 | 0.009908 | 0.0034254 | 0.0041577 | 0.0030155 | 0.0024732 | 0.002676 | 0.0029182 | 0.0021221 | 0.003506 | 0.0029813 | 0.0018591 |
| K04561 | norB | 0.0200605 | 0.0195881 | 0.0095223 | 0.0131056 | 0.0144974 | 0.0071523 | 0.0068179 | 0.0080425 | 0.0067182 | 0.0075763 | 0.0148131 | 0.0118215 | 0.0117544 | 0.0128301 | 0.0121288 |
| K01501 | nitrilase | 0.0134194 | 0.0121328 | 0.0071798 | 0.0096789 | 0.0137304 | 0.0161955 | 0.0142433 | 0.0134872 | 0.0119834 | 0.0138943 | 0.0123726 | 0.0098609 | 0.0069694 | 0.0075642 | 0.0075319 |
| K15864 | nirS | 0.0119412 | 0.0125319 | 0.0206154 | 0.0280921 | 0.0216681 | 0.0308338 | 0.0308097 | 0.0290047 | 0.0312097 | 0.0333701 | 0.0262427 | 0.0280145 | 0.0301779 | 0.0290567 | 0.0332514 |
| K00368 | nirK | 0.0108423 | 0.0083646 | 0.0191373 | 0.0138356 | 0.0077917 | 0.0089279 | 0.009196 | 0.0060704 | 0.0054399 | 0.0054736 | 0.0008349 | 0.0019595 | 0.0024416 | 0.0033008 | 0.0028367 |
| K00363 | nirD | 0.0042731 | 0.005948 | 0.0081141 | 0.0104129 | 0.0133877 | 0.0028307 | 0.0019593 | 0.0026841 | 0.001615 | 0.0019849 | 0.0033834 | 0.0041305 | 0.0030825 | 0.0032291 | 0.0018469 |
| K00362 | nirB | 0.0201818 | 0.024923 | 0.0153337 | 0.0251677 | 0.0434154 | 0.0054113 | 0.0075522 | 0.0122833 | 0.0075673 | 0.0081763 | 0.020412 | 0.0166067 | 0.013127 | 0.0098158 | 0.0057778 |
| K00366 | nirA | 0.0016891 | 0.0012049 | 0.0019878 | 0.0030871 | 0.005115 | 0.0020974 | 0.0011636 | 0.0013683 | 0.001788 | 0.0017412 | 0.0011856 | 0.0014154 | 0.0029456 | 0.0013397 | 0.001743 |
| K02591 | nifK | 0.0015708 | 0.0020149 | 0 | 0.00032 | 0.0058248 | 0.0001557 | 0.0003243 | 0.0002425 | 0.0002344 | 0.0004769 | 0.0048527 | 0.0002243 | 0.0005323 | 0.000209 | 0.0002611 |
| K02588 | nifH | 0.0011037 | 0.0021214 | 0.0007606 | 0.0006059 | 0.0032594 | 0.0008106 | 0.000787 | 0.0005934 | 0.001394 | 0.0010873 | 0.0029483 | 0.0009272 | 0.0013741 | 0.0009554 | 0.0010135 |
| K02586 | nifD | 0.0014676 | 0.0028859 | 0.0002603 | 0.0005159 | 0.0036705 | 6.161E-05 | 0.0002822 | 0.0002004 | 0.0003839 | 0.0004935 | 0.0048204 | 9.135E-05 | 0.0005664 | 0.0003395 | 0.0002199 |
| K00459 | ncd2 | 0.0360884 | 0.0390183 | 0.0295279 | 0.0375581 | 0.0370996 | 0.036169 | 0.0398217 | 0.0420767 | 0.0391431 | 0.0405016 | 0.0355548 | 0.0437904 | 0.0368218 | 0.0316012 | 0.0355745 |
| K00372 | nasA | 0.0106968 | 0.0144834 | 0.0186283 | 0.0176851 | 0.0311538 | 0.0036468 | 0.0037082 | 0.004901 | 0.0020099 | 0.0030365 | 0.006617 | 0.0048282 | 0.0053361 | 0.0033823 | 0.0014248 |
| K00374 | narI | 0.0086164 | 0.0104492 | 0.0079463 | 0.0090518 | 0.0119354 | 0.0044985 | 0.0042329 | 0.0042046 | 0.0043027 | 0.0042683 | 0.0058185 | 0.0055517 | 0.0054805 | 0.0065598 | 0.0049182 |
| K00371 | narH | 0.0188886 | 0.0195496 | 0.0367793 | 0.0262149 | 0.015182 | 0.0139637 | 0.012623 | 0.0136726 | 0.0134361 | 0.0143198 | 0.01581 | 0.019012 | 0.0185266 | 0.018628 | 0.0214859 |
| K00370 | narG | 0.041488 | 0.040777 | 0.0920164 | 0.0681499 | 0.0254616 | 0.0322561 | 0.0323501 | 0.0309669 | 0.0312844 | 0.0337515 | 0.0318254 | 0.0474092 | 0.0492195 | 0.0540521 | 0.0583569 |
| K00376 | narB | 0.0195228 | 0.0176561 | 0.0244003 | 0.0298023 | 0.0285702 | 0.026888 | 0.0260615 | 0.0283113 | 0.0309883 | 0.0298626 | 0.0313029 | 0.0397541 | 0.0433855 | 0.0450672 | 0.0431482 |
| K02568 | napB | 0.0080834 | 0.0079931 | 0.0039211 | 0.004929 | 0.0115662 | 0.0063491 | 0.0073523 | 0.0095906 | 0.010538 | 0.0089639 | 0.0128795 | 0.0128866 | 0.0130554 | 0.0107388 | 0.0142183 |
| K02567 | napA | 0.0265652 | 0.0268547 | 0.0175949 | 0.027976 | 0.0310814 | 0.0415458 | 0.0432897 | 0.0472557 | 0.0526095 | 0.0551505 | 0.0639003 | 0.0654996 | 0.062785 | 0.0630628 | 0.0634695 |
| K05601 | hcp | 0.0158272 | 0.0164763 | 0.0009632 | 0.0041706 | 0.0144243 | 0.0076361 | 0.005905 | 0.0091494 | 0.0106639 | 0.0103061 | 0.031806 | 0.0167349 | 0.0141563 | 0.0131231 | 0.0140334 |
| K10535 | hao | 8.095E-05 | 5.749E-05 | 0.0038492 | 0.0068883 | 0.0015042 | 0.0009355 | 0.0006234 | 0.0005387 | 0.0002464 | 0.0012829 | 0.0003547 | 0.0003834 | 0.0001879 | 0.0003024 | 0.0004641 |
| K00260 | gudB | 0.0039108 | 0.0027592 | 0.0031667 | 0.0020516 | 0.0008078 | 0.0013555 | 0.0005197 | 0.0006523 | 0.0007631 | 0.0006985 | 0.00118 | 0.0007204 | 0.0005308 | 0.0009741 | 0.0004731 |
| K00284 | gltS | 0.0223314 | 0.0168917 | 0.0273691 | 0.0200922 | 0.0078283 | 0.025389 | 0.0201248 | 0.0201449 | 0.0191308 | 0.0164402 | 0.0146047 | 0.0149303 | 0.013159 | 0.0146364 | 0.0147251 |
| K00266 | gltD | 0.118502 | 0.1078818 | 0.0924191 | 0.0922476 | 0.0710372 | 0.0794652 | 0.0745593 | 0.0800277 | 0.0763916 | 0.0762696 | 0.0823725 | 0.0728382 | 0.065578 | 0.0692696 | 0.0689228 |
| K00265 | gltB | 0.0724641 | 0.0850458 | 0.1091855 | 0.1120692 | 0.0942355 | 0.114044 | 0.1170358 | 0.109875 | 0.1215377 | 0.117557 | 0.097649 | 0.1015099 | 0.1042641 | 0.1082205 | 0.1088225 |
| K00264 | GLT1 | 0.0003254 | 0.0003108 | 0.0001924 | 0.0004759 | 0.0007484 | 0.0013703 | 0.0016052 | 0.0009654 | 0.0010853 | 0.0016776 | 0.0003261 | 0.0013612 | 0.0014276 | 0.0009049 | 0.001108 |
| K01915 | glnA | 0.1678909 | 0.1530066 | 0.1547714 | 0.1378721 | 0.149405 | 0.1758589 | 0.1818505 | 0.171496 | 0.1714065 | 0.1711386 | 0.1526747 | 0.1653946 | 0.1720121 | 0.1770336 | 0.1793574 |
| K00261 | gdhA | 0.0769075 | 0.0644571 | 0.0546335 | 0.0480252 | 0.0479128 | 0.081372 | 0.0824908 | 0.0780428 | 0.0828707 | 0.0762146 | 0.0652711 | 0.0718959 | 0.0665682 | 0.0685528 | 0.0676836 |
| K00262 | gdhA | 0.0406829 | 0.0370553 | 0.0181584 | 0.0154609 | 0.0246701 | 0.0370717 | 0.0347816 | 0.0404805 | 0.0383633 | 0.0373679 | 0.05166 | 0.0419001 | 0.0384068 | 0.0373991 | 0.0347655 |
| K15371 | GDH2 | 0.048945 | 0.0528947 | 0.0640983 | 0.0496228 | 0.0643134 | 0.0944774 | 0.0962223 | 0.0904155 | 0.0892571 | 0.0918925 | 0.0636111 | 0.0706336 | 0.0839825 | 0.0794817 | 0.0731892 |
| K01455 | E3.5.1.49 | 0.004441 | 0.0059056 | 0.0043794 | 0.0028715 | 0.0051338 | 0.0135546 | 0.0127149 | 0.012719 | 0.0090535 | 0.0113935 | 0.0082114 | 0.0087481 | 0.0122335 | 0.0102673 | 0.0125456 |
| K01673 | cynT | 0.0440639 | 0.0444203 | 0.0384895 | 0.0438505 | 0.0412378 | 0.042319 | 0.0414454 | 0.0396411 | 0.037824 | 0.0370623 | 0.0299206 | 0.0360129 | 0.0350583 | 0.0381473 | 0.034784 |
| K01725 | cynS | 0.0048337 | 0.0059555 | 0.0020997 | 0.0012257 | 0.0068052 | 0.001915 | 0.0012245 | 0.0018406 | 0.0011753 | 0.0009108 | 0.0001805 | 8.54E-05 | 0 | 0 | 0 |
| K01674 | cah | 0.0056039 | 0.00599 | 0.002139 | 0.0063939 | 0.0036707 | 0.0050492 | 0.0049727 | 0.0052655 | 0.0084958 | 0.0073282 | 0.0076177 | 0.008058 | 0.0092246 | 0.0092952 | 0.0109068 |
| K00926 | arcC | 0.0429562 | 0.0358765 | 0.0244937 | 0.0255237 | 0.0188416 | 0.0331849 | 0.032706 | 0.0355549 | 0.0349088 | 0.0279891 | 0.0423722 | 0.0299109 | 0.0278955 | 0.0286437 | 0.0320965 |

Table S3. Network node taxonomic and modularity properties in this study.

| Nodes | Label | Phylum | Modularity class |
| --- | --- | --- | --- |
| OTU3405 | OTU3405 | Epsilonbacteraeota | module 4 |
| OTU3389 | OTU3389 | Proteobacteria | module 4 |
| OTU3045 | OTU3045 | Proteobacteria | module 4 |
| OTU3123 | OTU3123 | Fusobacteria | module 4 |
| OTU2069 | OTU2069 | Bacteroidetes | module 2 |
| OTU3249 | OTU3249 | Cyanobacteria | module 3 |
| OTU57 | OTU57 | Epsilonbacteraeota | module 2 |
| OTU3547 | OTU3547 | Nitrospirae | module 3 |
| OTU1690 | OTU1690 | Proteobacteria | module 2 |
| OTU487 | OTU487 | Chloroflexi | module 2 |
| OTU376 | OTU376 | Proteobacteria | module 2 |
| OTU1447 | OTU1447 | Proteobacteria | module 2 |
| OTU1513 | OTU1513 | Proteobacteria | module 2 |
| OTU3299 | OTU3299 | Firmicutes | module 3 |
| OTU2295 | OTU2295 | Proteobacteria | module 1 |
| OTU2142 | OTU2142 | Cyanobacteria | module 1 |
| OTU2444 | OTU2444 | Epsilonbacteraeota | module 1 |
| OTU2858 | OTU2858 | Planctomycetes | module 1 |
| OTU1830 | OTU1830 | Proteobacteria | module 2 |
| OTU2787 | OTU2787 | Proteobacteria | module 1 |
| OTU2977 | OTU2977 | Proteobacteria | module 1 |
| OTU2525 | OTU2525 | Proteobacteria | module 1 |
| OTU2612 | OTU2612 | Proteobacteria | module 1 |
| OTU2516 | OTU2516 | Chloroflexi | module 1 |
| OTU1774 | OTU1774 | Bacteroidetes | module 2 |
| OTU3159 | OTU3159 | Firmicutes | module 3 |
| OTU238 | OTU238 | Proteobacteria | module 1 |
| OTU2414 | OTU2414 | Proteobacteria | module 1 |
| OTU2566 | OTU2566 | Nitrospirae | module 1 |
| OTU2422 | OTU2422 | Nitrospirae | module 1 |
| OTU2230 | OTU2230 | Proteobacteria | module 1 |
| OTU2679 | OTU2679 | Proteobacteria | module 1 |
| OTU1839 | OTU1839 | Nitrospirae | module 2 |
| OTU2339 | OTU2339 | Proteobacteria | module 1 |
| OTU2782 | OTU2782 | Chloroflexi | module 1 |
| OTU1916 | OTU1916 | Verrucomicrobia | module 2 |
| OTU3351 | OTU3351 | Epsilonbacteraeota | module 1 |
| OTU2664 | OTU2664 | Proteobacteria | module 1 |
| OTU3226 | OTU3226 | Proteobacteria | module 3 |
| OTU2611 | OTU2611 | Proteobacteria | module 1 |
| OTU3340 | OTU3340 | Proteobacteria | module 1 |
| OTU3555 | OTU3555 | Patescibacteria | module 1 |
| OTU2457 | OTU2457 | Firmicutes | module 1 |
| OTU2112 | OTU2112 | Proteobacteria | module 1 |
| OTU2707 | OTU2707 | Proteobacteria | module 3 |
| OTU3181 | OTU3181 | Proteobacteria | module 3 |
| OTU1795 | OTU1795 | Proteobacteria | module 2 |
| OTU3472 | OTU3472 | Proteobacteria | module 3 |
| OTU3116 | OTU3116 | Proteobacteria | module 3 |
| OTU3127 | OTU3127 | Proteobacteria | module 3 |
| OTU3301 | OTU3301 | Proteobacteria | module 3 |
| OTU3293 | OTU3293 | Proteobacteria | module 3 |
| OTU2155 | OTU2155 | Actinobacteria | others |
| OTU2796 | OTU2796 | Nitrospirae | module 5 |
| OTU3022 | OTU3022 | Proteobacteria | module 5 |
| OTU2186 | OTU2186 | Proteobacteria | module 4 |
| OTU771 | OTU771 | Chloroflexi | module 2 |
| OTU871 | OTU871 | Proteobacteria | module 2 |
| OTU650 | OTU650 | Proteobacteria | module 2 |
| OTU2984 | OTU2984 | Proteobacteria | module 3 |
| OTU2781 | OTU2781 | Proteobacteria | module 3 |
| OTU3264 | OTU3264 | Proteobacteria | module 1 |
| OTU3241 | OTU3241 | Spirochaetes | module 3 |
| OTU3585 | OTU3585 | Rokubacteria | module 3 |
| OTU2415 | OTU2415 | Proteobacteria | module 3 |
| OTU1443 | OTU1443 | Proteobacteria | module 2 |
| OTU1471 | OTU1471 | Proteobacteria | module 2 |
| OTU3519 | OTU3519 | Proteobacteria | module 3 |
| OTU1767 | OTU1767 | Chloroflexi | module 2 |
| OTU2986 | OTU2986 | Proteobacteria | module 3 |
| OTU1838 | OTU1838 | Proteobacteria | module 2 |
| OTU2662 | OTU2662 | Proteobacteria | module 3 |
| OTU1933 | OTU1933 | Actinobacteria | module 6 |
| OTU2084 | OTU2084 | Proteobacteria | module 6 |
| OTU1728 | OTU1728 | Proteobacteria | module 2 |
| OTU1645 | OTU1645 | Proteobacteria | module 2 |
| OTU3525 | OTU3525 | Proteobacteria | module 3 |
| OTU3201 | OTU3201 | Chloroflexi | module 3 |
| OTU3323 | OTU3323 | Proteobacteria | module 3 |
| OTU21 | OTU21 | Firmicutes | module 2 |
| OTU3407 | OTU3407 | Proteobacteria | module 1 |
| OTU1533 | OTU1533 | Proteobacteria | module 2 |
| OTU2081 | OTU2081 | Acidobacteria | module 2 |
| OTU942 | OTU942 | Proteobacteria | module 2 |
| OTU167 | OTU167 | Proteobacteria | others |
| OTU2383 | OTU2383 | Actinobacteria | others |
| OTU2188 | OTU2188 | Proteobacteria | others |
| OTU2194 | OTU2194 | Actinobacteria | module 3 |
| OTU2777 | OTU2777 | Proteobacteria | module 1 |
| OTU2185 | OTU2185 | Bacteroidetes | others |
| OTU394 | OTU394 | Bacteroidetes | others |
| OTU2065 | OTU2065 | Firmicutes | others |
| OTU2274 | OTU2274 | Actinobacteria | others |
| OTU1491 | OTU1491 | Proteobacteria | module 1 |
| OTU2061 | OTU2061 | Proteobacteria | others |
| OTU2181 | OTU2181 | Actinobacteria | others |
| OTU2071 | OTU2071 | Actinobacteria | others |
| OTU2115 | OTU2115 | Bacteroidetes | others |
| OTU2397 | OTU2397 | Bacteroidetes | others |
| OTU2453 | OTU2453 | Chloroflexi | others |

The other indicates modules with an abundance of less than 2%
